# Supplementary material for: Talk the talk and walk the walk. Evaluation of autonomy in aging and Alzheimer disease by simulating instrumental activities of daily living: the S-IADL
Source: PeerJ. 2016 Sep 13;4:e2351. doi: 10.7717/peerj.2351 (PMC5028723; doi:10.7717/peerj.2351)
Supplement: Appendix S1 [file peerj-04-2351-s002.docx]

| **ANCOVA Tests of Between-Subjects Effects** | | | | | | |
| --- | --- | --- | --- | --- | --- | --- |
| **Dependent Variable: IADL** | | | | | | |
| Source | Type III Sum of Squares | df | Mean Square | F | Sig. | Partial Eta Squared |
| Corrected Model | 4552.547^a^ | 3 | 1517.516 | 23.567 | .000 | .472 |
| Intercept | 11289.168 | 1 | 11289.168 | 175.322 | .000 | .689 |
| Group | 1544.176 | 1 | 1544.176 | 23.981 | .000 | .233 |
| Gender | 299.367 | 1 | 299.367 | 4.649 | .034 | .056 |
| Gpe * Gender | 380.203 | 1 | 380.203 | 5.905 | .017 | .070 |
| Error | 5086.891 | 79 | 64.391 |  |  |  |
| Total | 106065.943 | 83 |  |  |  |  |
| Corrected Total | 9639.438 | 82 |  |  |  |  |
| a. R Squared = .472 (Adjusted R Squared= .452) | | | | | | |

|  | | | | | | |
| --- | --- | --- | --- | --- | --- | --- |
| **Dependant Variable: SIADL** | | | | | | |
| Source | Type III Sum of Squares | df | Mean Square | F | Sig. | Partial Eta Squared |
| Corrected Model | 4261.894^a^ | 3 | 1420.631 | 12.936 | .000 | .329 |
| Intercept | 1976.916 | 1 | 1976.916 | 18.001 | .000 | .186 |
| Group | 767.189 | 1 | 767.189 | 6.986 | .010 | .081 |
| Gender | 3.409 | 1 | 3.409 | .031 | .861 | .000 |
| Group * Gender | 43.703 | 1 | 43.703 | .398 | .530 | .005 |
| Error | 8675.818 | 79 | 109.820 |  |  |  |
| Total | 26773.304 | 83 |  |  |  |  |
| Corrected Total | 12937.712 | 82 |  |  |  |  |
| a. R Squared = .329 (Adjusted R Squared = .304) | | | | | | |

**ANCOVA Tests of Between-Subjects Effects**

|  | | | | | | |
| --- | --- | --- | --- | --- | --- | --- |
| **Dependent Variable: IADL** | | | | | | |
| Source | Somme des carrés de type III | ddl | Moyenne des carrés | D | Sig. | Eta au carré partiel |
| Corrected Model | 5008,475^a^ | 2 | 2504,237 | 43,261 | ,000 | ,520 |
| Intercept | 14,603 | 1 | 14,603 | ,252 | ,617 | ,003 |
| Age | 865,574 | 1 | 865,574 | 14,953 | ,000 | ,157 |
| Group | 1650,883 | 1 | 1650,883 | 28,519 | ,000 | ,263 |
| Error | 4630,963 | 80 | 57,887 |  |  |  |
| Total | 106065,943 | 83 |  |  |  |  |
| CorrectedTotal | 9639,438 | 82 |  |  |  |  |
| a. R Squared = ,520 (Adjusted R Squared = ,508) | | | | | | |

|  | | | | | | |
| --- | --- | --- | --- | --- | --- | --- |
| **Dependent Variable: SIADL** | | | | | | |
| Source | Somme des carrés de type III | ddl | Moyenne des carrés | D | Sig. | Eta au carré partiel |
| Corrected Model | 6151,192^a^ | 2 | 3075,596 | 36,255 | ,000 | ,475 |
| Intercept | 787,991 | 1 | 787,991 | 9,289 | ,003 | ,104 |
| Age | 1946,501 | 1 | 1946,501 | 22,945 | ,000 | ,223 |
| Group | 1129,379 | 1 | 1129,379 | 13,313 | ,000 | ,143 |
| Error | 6786,520 | 80 | 84,831 |  |  |  |
| Total | 26773,304 | 83 |  |  |  |  |
| Corrected Total | 12937,712 | 82 |  |  |  |  |
| a. R Squared = ,475 (Adjusted R Squared = ,462) | | | | | | |
